# Social Media Use and Digital Professionalism in Nursing Practice: Cross-Sectional Questionnaire Study

**DOI:** 10.2196/92923

**Published:** 2026-10-21

**Authors:** Melita Peršolja, Anton Grmšek Svetlin

**Affiliations:** 1 Faculty of Health Sciences, University of Primorska, Polje 42, Izola, Izola-Isola, 6310, Slovenia, 386 31873515; 2 Izola General Hospital, Izola, Izola-Isola, Slovenia

**Keywords:** nursing, hospitals, digitalization, social media, informatics

## Abstract

**Background:**

The rapid growth of social media has transformed communication, with more than 5.66 billion users worldwide in 2025 who spent an average of 141 minutes daily on platforms such as Facebook, Instagram, WhatsApp, YouTube, and TikTok. Nurses frequently use social media to exchange work-related information, communicate with others, and relieve stress. While social media can support professional development, excessive or inappropriate use at work may compromise patient care. Despite widespread use, research on nurses’ demographic factors and actual workplace social media behaviors remains limited.

**Objective:**

The aim of the study was to determine how nurses use social media at work and how their demographic characteristics (eg, gender, age, and number of years working in health care) are related to the use of social media at work.

**Methods:**

This study used a quantitative, descriptive, exploratory, and nonexperimental empirical research design. Data were collected via a survey administered to a convenience sample of 132 nurses. The sample comprised of 98 women (74.2%) and 34 men (25.8%). The questionnaire consisted of 17 statements related to social media use in the workplace. A statistical significance level of *P*<.05 was applied. Descriptive statistics and inferential statistical tests were used.

**Results:**

Nurses used Facebook the most and spent between 45 minutes and 1 hour per day on social media. Study participants reported that they primarily used social media for communication with colleagues and acquiring new knowledge. They also used social media for its technical functions, such as file sharing. Nevertheless, a substantial number of nurses reported using social media during working hours, and many had experienced creating an uncomfortable situation after declining social media interactions with patients or colleagues.

**Conclusions:**

This study highlights the important topic of social media use at work by nurses. The study found that nurses frequently used social media, primarily for professional communication and knowledge acquisition. While most demonstrated positive use patterns, the findings indicate that social media use during work may pose potential challenges to professionalism. These results highlight the need for clear guidelines and training to ensure that nurses use social media responsibly, maintaining both professional boundaries and personal integrity. Encouraging mindful and regulated use can enhance the benefits of social media while minimizing risks in the health care setting.

## Introduction

We are witnessing rapid technological development in the 21st century, particularly in relation to the internet and social media. Social media is defined as a group of online applications that enable the creation and sharing of content generated by users [1]. More than a decade ago, Kaplan and Haenlein [2] defined five main categories of social media: (1) collaborative projects (eg, Wikipedia), (2) blogs or microblogs (eg, X), (3) content communities (eg, YouTube), (4) social networking sites (eg, Instagram and Facebook), and (5) virtual gaming worlds (eg, Second Life).

Unlike traditional media, where users are usually passive recipients of information, social media platforms enable individuals to actively participate in creating and sharing content with others, which is precisely why they are becoming an increasingly important means of communication and connection among people [3]. In recent years, the use of social media has thus become an indispensable part of everyday life for many around the world. Many people spend several hours a day on platforms such as Facebook, Messenger, and Instagram, whether to maintain contacts, exchange information, or for entertainment and relaxation [4]. The data show that the number of social media users worldwide had reached 5.66 billion in October 2025, or approximately 70.8% of the global population [5]. Statistics indicate that in 2025, users spent an average of 141 minutes per day on social media. Compared with data from 2015, this number is on the rise, as daily social media use at that time was just 111 minutes [6]. The data further reveal that the most frequently used social media platforms are Facebook (Meta Platforms), Instagram (Meta Platforms), WhatsApp (Meta Platforms), YouTube (Alphabet Inc), and TikTok (ByteDance) [7].

According to research by Lefebvre et al [8], 87% of the 397 nurse respondents had an active user account on at least one social media platform. It was also found that older age is associated with less frequent use of social media. A study conducted by Wang et al [9] using a sample of 658 nurses revealed that approximately 56% of participants spend between 1 and 3 hours per day using social media. The majority (67.2%) reported that they use social media to exchange work-related information with colleagues. At the same time, 50.3% stated that they had witnessed inappropriate posts by their colleagues. In their study of a sample of 43 nurses at the tertiary health care level, Al-Rumhi et al [10] found that all the participants used social media, most commonly WhatsApp. They also found that most nurses spend 1 to 2 hours a day on social media. The authors state that the main motives for nurses to use social media were to acquire knowledge, communicate, and establish contact with patients, thus reducing the number of hospital visits.

To maintain professional integrity and ensure the responsible use of social media, the American Nurses Association has issued the following recommendations for its use [11]: (1) maintain professionalism online, (2) avoid sharing patient posts, (3) exercise caution when posting photos from the workplace, (4) avoid establishing contact with patients and their families, (5) refrain from disparaging one’s employing organization, and (6) avoid posts that cast the nursing profession in a negative light. The growing use of social media in health care has highlighted the importance of digital professionalism, defined as the expression of professional values and behaviors in digital environments. As nurses increasingly engage with social media, understanding how these platforms are used in workplace contexts has become an important aspect of maintaining professional boundaries and ethical practice [12-14].

Through work, people satisfy numerous needs—biological, social, economic, and psychological—including the need for success, belonging, recognition, respect, and self-fulfillment. At the same time, work involves a system of social interactions that shape people, connect them with others, and significantly influence their quality of life [15,16]. Nurses who are exposed to high workloads are much more prone to burnout, absenteeism, and errors in patient care [17]. This is why mobile phones and social media are digital tools that health care workers often use in their work to reduce stress and divert their attention from work-related burdens [8,18]. However, excessive use of social media during working hours can compromise the quality of patient care [19] because it can reduce the time and energy devoted to patient-focused tasks, thereby reducing their work performance [20].

Since nurses are frequent users of social media [10,18], it is crucial that they recognize the associated dangers, especially when it comes to using it at work. Although evidence from numerous countries shows that nurses frequently use social media, there is still limited research examining how demographic factors relate to its use within the nursing profession, and there is a lack of data describing how such behaviors occur and what factors influence them. Moreover, most of the existing research focuses on general attitudes and perceptions, whereas few studies offer empirical quantitative data on actual patterns of social media use by nurses at work. Additionally, research from many regions focuses primarily on nursing students rather than practicing nurses, leaving a knowledge gap about the experiences of nurses already engaged in direct patient care.

The purpose of this research was to describe social media use among nurses. The specific aim of the study was to examine how nurses use social media in the workplace and to explore how their demographic characteristics (eg, gender, age, and years of experience in health care) are associated with social media use at work. We thus set the following research questions: Which social media platforms do nurses use most frequently? How much time do nurses spend on social media each day? For what purposes do nurses use social media at work? How are the demographic characteristics of nurses related to their use of social media at work?

## Methods

A descriptive, exploratory, and nonexperimental method of empirical research was used with a survey technique.

### Research Instrument Description

The research instrument was developed by Landers and Callan [21]. To ensure conceptual and linguistic accuracy in the Slovenian context, the original English instrument was translated independently by 4 translators. Each translated version was subsequently back-translated into English and compared with the original instrument to identify potential discrepancies and ensure semantic consistency. The translated versions were reviewed and discussed by the research team, and any differences were resolved through consensus.

The questionnaire assessed work-related social media use broadly, including behaviors and interactions connected to the workplace context. It comprised 17 items rated on a 5-point Likert scale ranging from 1 (disagree) to 5 (agree), organized into 2 conceptually distinct subscales. The first subscale assessed the positive, purposeful dimensions of work-related social media use and contained 8 items (eg, “I use social media to learn how to do my job better” and “I use social media to contact colleagues when I cannot reach them in any other way”). The second subscale consisted of 9 items measuring negative or problematic social media behaviors in the workplace (eg, “My coworkers have been offended by something I posted on social media” and “I discuss negative feelings about patients or colleagues on social media”).

Before computing the composite work-related social media use score, all 9 items from the negative-behavior subscale were reverse-scored to ensure directional consistency across all 17 items. The composite score was then calculated as the mean of all items, with higher scores reflecting greater overall work-related social media use. Although the questionnaire originally consisted of 2 conceptually distinct subscales (beneficial work-related use and potentially harmful workplace behaviors), the overall composite score was calculated as the mean of all items to capture a broader continuum of overall work-related social media behavior. This approach is consistent with the conceptual framework proposed by Landers and Callan [21], where work-related social media use may range from professional and beneficial engagement to problematic or inappropriate workplace behaviors. Therefore, higher composite scores reflected a more favorable overall pattern of work-related social media use.

Internal consistency was satisfactory for both subscales, with a Cronbach α of 0.73 for the positive-purpose subscale and 0.74 for the negative-behavior subscale [22].

The third section of the questionnaire collected demographic data, including gender, age, years of nursing work experience, level of health care, and field of employment. Participants were additionally asked whether they held a management position, which social media platforms they used most frequently, and their average daily time spent on social media.

Prior to the main study, the Slovenian version of the questionnaire was pilot-tested among 20 nurses to evaluate clarity, comprehensibility, and cultural appropriateness. Minor linguistic adjustments were made based on participant feedback before the final version was distributed to the study sample. The responses obtained from the pilot testing were not included in the final analysis of this study.

### Research Sample Description

The target population of this study consisted of nurses employed in clinical practice across different health care settings in Slovenia. Eligible participants were individuals currently employed in nursing or direct health care practice who were actively involved in patient care or clinical work environments. To be included in the study, participants had to be currently employed within the health care system and willing to voluntarily complete the online questionnaire. Individuals not currently employed in clinical health care practice, health care students, retired health care workers, and respondents who did not complete the questionnaire in full were excluded from the final analysis.

A convenience sample was used in this study, and recruitment was conducted through snowball sampling, with the survey completed and distributed via online communication channels and social media. The snowball sampling approach was chosen because the study aimed to recruit nurses working across different health care settings in Slovenia, while online recruitment allowed efficient access to geographically dispersed participants. The survey link was initially distributed through professional and personal online communication channels, including social media platforms commonly used by health care professionals. Participants who accessed the questionnaire were subsequently encouraged to share the survey link with other nurses within their professional networks, thereby enabling broader recruitment through peer-to-peer distribution. To reduce potential sampling bias, the survey was distributed through multiple communication pathways (eg, social media platforms, email communication, professional networks, and direct person-to-person sharing) rather than relying on a single online platform, allowing access to nurses from different health care institutions and professional backgrounds.

The introductory page of the online questionnaire was viewed by 303 people. A total of 145 respondents began completing the questionnaire, and we included the responses of 132 respondents who completed the entire online questionnaire in our analysis. The research included 98 (74.2%) women and 34 (25.8%) men. The respondents were on average aged 34.3 (SD 10.33) years. The youngest respondent was aged 19 years and the oldest was 60 years. A total of 17 (12.9%) respondents were employed in primary health care, 88 (66.7%) in secondary health care, and 27 (20.5%) in tertiary health care. Most respondents were employed in hospital wards, for example, surgical (n=33, 25%), internal medicine and pediatrics (n=14, 10.6%), and intensive care (n=13, 9.8%). Respondents who did not select one of the offered work areas most often cited emergency medical services, radiology, and oncology as their areas of expertise.

Twenty-eight (21.2%) respondents had completed secondary education, 86 (65.2%) had completed higher education, 17 (12.9%) had completed postgraduate master’s education, and 1 (0.8%) respondent had obtained a doctorate. Most nurses (n=92, 69.7%) who participated in our study are employed as registered nurses. Thirty-one (23.5%) respondents stated that they performed a managerial function, while 101 (76.5%) respondents stated that they did not.

The largest group of nurses had been employed in health care for 1 to 4 years (n=26), followed by those employed for 20 years or more (n=25).

### Description of the Research Process and Data Processing

The survey was conducted using the open-source online survey application 1KA (version 26.09.01; Faculty of Social Sciences, Ljubljana, Slovenia). The questionnaire was distributed via online media and other communication channels using a snowball sampling method. Respondents had the opportunity to complete the questionnaire from July 9, 2025, to November 9, 2025.

Only fully completed questionnaires were included in the data analysis. The data were analyzed using SPSS (version 26.0; IBM Corp). Basic descriptive statistics were calculated (frequency, range, mean, and SD), and statistical tests were performed: the Kolmogorov-Smirnov test and 1-way ANOVA, independent-samples *t* test, 1-sample *t* test and the Tukey post hoc test. The level of statistical significance was set at *P*<.05. During the analysis, we created a composite variable, “Use of social media at work,” which combined all 17 statements about the use of social media at work into a single variable.

### Ethical Considerations

This study was conducted under Umbrella Ethical Approval for Research granted by the University of Primorska (approval 4264-19-6/23). Before completing the questionnaire, respondents gave their consent to participate in the survey. All procedures in this research were conducted in accordance with ethical guidelines outlined by the Declaration of Helsinki [23] and followed institutional protocols to ensure quality, integrity, and ethical responsibility.

## Results

### Overview

In our study, we found that Facebook was the most frequently used social media platform (n=113), followed by Instagram (n=105), Viber (Rakuten Viber; n=61), TikTok (n=55), and WhatsApp (n=34). Respondents who did not select one of the options provided most frequently cited LinkedIn (Microsoft), Pinterest (Pinterest, Inc), and BeReal (Voodoo).

Most respondents (45/132, 34.1%) spend between 45 minutes and 1 hour per day on social media, followed by those who use social media from 1 to 2 hours a day (37/132, 28%), 30 to 45 minutes a day (23/132, 17.4%), more than 2 hours a day (20/132, 15.2%), and less than 30 minutes (7/132, 5.3%).

The highest average value in the first set of questions (4.11, SD 1.09) was obtained for the statement that nurses use social media to contact colleagues when they cannot reach them in any other way. This indicates that one of the most important aspects of social media use is communication with colleagues. Nurses also rated relatively highly (mean 2.98, SD 1.24) the statement that they use social media to learn how to do their job better, which means that one of the purposes of using social media is education. In this section, they also expressed a high level of agreement (mean 2.95, SD 1.31) with the statement that they use the technical functions of social media, such as file sharing, to perform their work tasks, which reveals an additional aspect of their social media use. The lowest average value in this section of the questionnaire was for the statement that the respondents establish contact with potential new patients via social media (mean 1.40, SD 0.84). The average beta coefficient for the variability of the first set of statements was β=0.48. The full sociodemographic characteristics of the respondents are shown in Table 1.

The statements in the second set of questions mostly describe negative situations related to the use of social media at work, which means that lower average values for the statements indicate a lower level of agreement with the statements offered, suggesting less problematic use of social media. Nurses rated the statements in this section of the questionnaire low on average. Nevertheless, 2 statements received higher ratings, namely that nurses used social media when they should have been working (mean 3.08, SD 1.27) and that they had caused an unpleasant situation in the past by refusing to connect with colleagues, superiors, or patients via social networks (mean 2.75, SD 1.41). The lowest rating (mean 1.22, SD 0.59) was given to the statement that patients had posted information about respondents on social media that damaged their reputation at work, which means that most respondents had not yet encountered this situation. In this part of the questionnaire, the average beta coefficient measuring the variability of the statements was β=0.43.

**Table 1.** Sociodemographic characteristics of the participants (N=132).

Characteristics	Participants, n (%)
Gender	
Man	34 (25.8)
Woman	98 (74.2)
Health care setting	
Primary level (eg, primary health center and nursing home)	17 (12.9)
Secondary level (eg, general hospital)	88 (66.7)
Tertiary level (eg, clinical center)	27 (20.5)
Area of work	
Surgical ward	33 (25)
Internal medicine ward	14 (10.6)
Pediatric ward	14 (10.6)
Gynecological ward	9 (6.8)
Specialist outpatient clinic	8 (6.1)
Primary care clinic	3 (2.3)
Nursing home	3 (2.3)
Home care nursing	5 (3.8)
Intensive care unit	13 (9.8)
Emergency room	7 (5.3)
Other (eg, radiology ward)	23 (17.4)
Education level	
High-school diploma	28 (21.2)
Bachelor’s degree	86 (65.2)
Master’s degree	17 (12.9)
Doctoral degree	1 (0.8)
Professional role	
Practical nurse	2 (1.5)
Professional nurse	19 (14.4)
Registered nurse	92 (69.7)
Other (eg, managers)	19 (14.4)
Years of experience in nursing	
<1	20 (15.2)
1-4	26 (19.7)
5-9	23 (17.4)
10-14	19 (14.4)
15-19	19 (14.4)
≥20	25 (18.9)

The 2 statements that had the biggest impact (β=0.61) on the variability of the entire questionnaire were that respondents use the technical functions of social media to perform work tasks and that they ask for help via social media when they encounter problems at work (Table 2). The *P* values for all 17 statements were obtained with a 1-sample 2-tailed *t* test.

**Table 2.** Values of statements related to the use of social media at work.

Statement	Score, mean (SD; range)	β^a^	*P* value
I use social media to contact my coworkers when I am unable to reach them by other means.	4.11 (1.09; 1-5)	0.49	<.001
If I find something on social media that could harm the reputation of my coworkers or our organization, I let people know.	3.61 (1.21; 1-5)	0.21	.01
I have used social media when I should have been working.	3.08 (1.27; 1-5)	0.54	<.001
I have used social media to learn how to perform better in my job.	2.98 (1.24; 1-5)	0.47	<.001
I have taken advantage of the technical features of social media (such as file sharing or scheduling functions) to accomplish work tasks.	2.95 (1.31; 1-5)	0.61	<.001
I have created an uncomfortable situation by refusing connections with coworkers, supervisors, or patients via social media.	2.75 (1.41; 1-5)	0.55	<.001
I access social media while I am doing other work.	2.38 (1.14; 1-5)	0.53	<.001
I request help from people on social media when I am having trouble solving a problem at work.	2.33 (1.19; 1-5)	0.61	<.001
I use my organization’s official social media presence to network.	2.32 (1.43; 1-5)	0.42	<.001
I communicate with patients via social media.	1.62 (1.04; 1-5)	0.50	<.001
Other people at work have been offended by something I posted on social media.	1.61 (1.07; 1-5)	0.40	<.001
Through social media, I have entered a personal relationship with a patient or a colleague that I should not have had.	1.53 (0.93; 1-4)	0.43	<.001
I have stolen information or other content from social media and used it as if it were my own work.	1.42 (0.81; 1-4)	0.31	<.001
I connect with potential new patients through social media.	1.40 (0.84; 1-4)	0.50	<.001
I have discussed negative feelings toward patients or coworkers on social media.	1.37 (0.77; 1-4)	0.34	<.001
I have done poor quality work using my organization’s social media accounts.	1.25 (0.57; 1-4)	0.46	<.001
Patients posted information about me on social media that harmed my reputation at work.	1.22 (0.59; 1-4)	0.28	<.001

a β: beta coefficient

### Inferential Statistics

Due to the normal distribution of the data, which we determined using the Kolmogorov-Smirnov test (Kolmogorov-Smirnov statistic=0.07; *P*=.20), we used parametric tests for further analysis.

Using the independent-samples *t* test, we did not find any statistically significant differences in the use of social media between genders (*t*_130_=0.177; *P*=.86; Cohen *d*=0.04). Additionally, we did not find statistically significant differences in the use of social media according to the managerial function of nurses (*t*_130_=–0.294; *P*=.77; Cohen *d*=–0.06).

A 1-way ANOVA showed statistically significant differences (*F*_2,129_=7.399; *P*=.001; η²=0.103) in the use of social media at work among nurses at the primary (mean 40.47, SD 7.61), secondary (mean 38.75, SD 8.44), and tertiary (mean 32.18, SD 8.08) levels of health care. The Tukey post hoc test showed statistically significant differences (*P*=.001) between nurses at the secondary and tertiary levels of health care. Nurses employed at the tertiary level achieved lower (more favorable) values, which indicated a more professional and thoughtful use of social media compared with nurses at the secondary level of health care.

By contrast, ANOVA did not detect statistically significant differences in the use of social media based on professional role (*F*_4,127_=1.268; *P*=.29; η²=0.03) and years of nursing experience (*F*_5,126_=0.883; *P*=.49; η²=0.03).

## Discussion

### Principal Findings

The purpose of our research was to describe the use of social media at work among nurses. We found that the most frequently used platform among the respondents was Facebook, with 113 nurses using it. Other frequently used social media platforms were Instagram, Viber, TikTok, and WhatsApp. Gamor et al [24] reported similar results, finding that health care workers most frequently used Facebook, WhatsApp, Twitter (now X), YouTube, and Instagram. In line with our results, Van Ravenswaay et al [25] found that Facebook was the most frequently used social media platform among health care professionals.

In our study, we found that nurses are frequent users of social media, using it for an average of 45 minutes to 1 hour per day. Surani et al [26] came to a similar conclusion, finding that most nurses spent between 31 and 60 minutes per day on social media. Ahmead et al [27] found that 33.5% of nurses spent more than 1 hour per day on social media, while 45% of them used social media for 1 to 3 hours per day. They also found that 76.6% of nurses spent more than 1 hour per day on social media at work. In our study, we found that nurses often used social media when they should have been working. The use of social media in the workplace can be potentially harmful to nurses’ work. Wilson [28] identified 5 key risks associated with the use of social media at work, namely (1) a decline in employee productivity, (2) information leaks, (3) damage to the institution’s reputation, (4) cybercrime, and (5) data loss due to insecure passwords. Geraghty et al [29] concluded that the use of social media at work can be detrimental to nurses, as it reduces their level of attention and perception of the situation at hand, which are both crucial for the safe care of patients.

We found that nurses primarily used social media to communicate with colleagues when they could not reach them by other means. This was also reflected in the more frequent use of social media platforms whose primary purpose is communication (eg, Viber and WhatsApp). Gamor et al [24] also found that one of the primary purposes of social media use for nurses was to communicate with colleagues. In addition, we found that nurses often used social media to learn how to do their jobs better. The results were consistent with the findings of other studies, which concluded that nurses often used social media for professional development, knowledge exchange, and education [24,30,31].

In the second part of the questionnaire, the respondents agreed least with the statement that they use social media to establish contact with patients. It is very important for nurses to be professional in their work, to have clear boundaries, and to maintain a professional relationship with their patients to protect their integrity. At the beginning of the rise of social media, Aylott [32] noted that the line between personal and professional relationships between nurses and patients was quickly blurred, especially with the help of social media, which facilitates communication and accessibility. Valente [33] notes that nurses may receive friend requests from patients on social media, but that crossing professional boundaries in this way can significantly damage their professionalism. At the same time, such behavior undermines patients’ trust in the work of nurses and damages the therapeutic relationship.

Because nurses often do not respond to friend or follow requests on social media or other calls to establish contact with patients or colleagues, they can quickly create an uncomfortable situation. Most of the participants in our study agreed that they had been in such a situation before. Unhjem [34] found that nurses often received friend requests from patients on Facebook, and that most nurses decided to accept them, even though this negatively affected their professionalism and integrity and represented a serious violation of the therapeutic relationship with the patient. The author concluded that most nurses accepted the friend requests because otherwise they felt uncomfortable and guilty about rejecting patients. Importantly, this discomfort is not merely interpersonal but is grounded in ethical and patient safety concerns. Professional standards emphasize that nurses must respect defined boundaries when interacting with patients, whether in clinical settings or on social media [35]. From an ethical and patient safety standpoint, several key risks are associated with social media interactions involving patients, such as the blurring of professional boundaries [34] and compromising confidentiality and privacy [36]. It is thus crucial for all nurses to maintain their professionalism outside the clinical settings where they work and not to enter into personal relationships with patients, thus preserving their professionalism.

In our study, we found that nurses employed at the tertiary level of health care used social media in the most professional manner. This could be attributed to clearer policies and guidelines in tertiary health care institutions, which encourage the professional use of social media in clinical practice [37]. This suggests that environments with more established governance provide a clearer framework for social media use that mitigates any potential risks (such as confidentiality concerns and unprofessional behavior). However, this study did not find any statistically significant differences in social media use based on age (*P*=.99), gender (*P*=.11), or job position (*P*=.29). In contrast, Nelson-Brantley et al [31] found that the use of social media differed statistically significantly among nurses based on demographic data such as age, job position, and type of employment. Lefebvre et al [8] found that age affected the frequency of social media use and that the use declined with increasing age. This could be attributed to the fact that younger nurses generally have easier and more frequent access to mobile phones.

This study contributes to the growing body of literature on digital professionalism and social media use in health care by providing empirical evidence on how nurses use social media within clinical work environments. While previous research has often focused on nursing students or general attitudes toward social media, our study offers insight into actual workplace behaviors among practicing nurses. The findings have relevance beyond the Slovenian health care setting, as health care institutions worldwide increasingly face challenges related to digital professionalism, maintaining professional boundaries, and ensuring the responsible use of social media in clinical practice. Our results thus contribute to the broader international discussion on how digital technologies are reshaping professional behavior within modern health care systems.

### Study Limitations and Suggestions for Future Research

While nurses in clinical practice participated in the study, the study relied on nonprobability sampling methods and included a small, uneven sample and spanned different health care settings, which limited the ability to generalize the findings to the broader global nursing workforce. Future research should therefore include a larger sample of nurses across more diverse regions of the world and systems to enable meaningful comparisons across different countries and contexts. Through multicountry comparative studies, researchers could examine how cultural norms affect nurses’ social media use in the workplace.

Another limitation of this study relates to the use of snowball sampling and online recruitment through social media and digital communication channels. Because participation depended on voluntary engagement with online distribution channels, the study may have disproportionately reached nurses who are more familiar with or more active users of social media. This may have introduced self-selection bias and potentially overrepresented participants with higher levels of digital engagement. Consequently, the findings cannot be generalized to the broader nursing population.

Another limitation is the low number of respondents holding master’s or doctoral degrees, which prevents the findings from being extended to highly educated nurses. In the future, it is imperative that more educated nurses participate in the study.

Another limitation of the study is that no a priori sample size calculation or power analysis was performed before data collection. Consequently, the adequacy of the sample size for detecting smaller effects cannot be fully determined and should be considered when interpreting the findings.

Furthermore, the cross-sectional design of the study limits the ability to draw causal conclusions regarding the relationships between demographic characteristics and social media use. The findings represent a snapshot of participants’ behaviors and perceptions at a single point in time. Moreover, data were collected using a self-report questionnaire, which may have introduced response bias and social desirability bias. Participants may have underreported potentially inappropriate social media behaviors or overreported professionally desirable practices, which could have influenced the accuracy of the reported findings.

Additional qualitative research is recommended using in-depth individual interviews or multiple focus groups to gain a more comprehensive understanding of how nurses use social media in clinical practice, and experimental research is also recommended. In this way, researchers could test how specific types of social media influence nurses’ decision-making and how they affect their work performance.

### Conclusions

Our study demonstrates that nurses in clinical practice are frequent users of social media, primarily for professional communication with colleagues and for acquiring new knowledge. However, our findings also highlight potential challenges, including the use of social media during work hours, which may distract nurses from patient care. It is essential that nurses maintain professional boundaries and adhere to ethical guidelines when using social media in the workplace, both to safeguard patient safety and to uphold the integrity of the nursing profession. By promoting responsible and purposeful social media use, health care institutions can support nurses in leveraging digital tools to enhance professional development, collaboration, and the overall quality of patient care.
